# A case of reccuring giant condyloma of vulva in infant without sexual abuse successfully treated with electrocoagulation in Benin

**DOI:** 10.11604/pamj.2017.27.159.11998

**Published:** 2017-06-30

**Authors:** Fabrice Akpadjan, Hugues Adégbidi, Cossi Angelo Attinsounon, Christiane Koudoukpo, Bérénice Dégboé, Nadège Agbessi, Félix Atadokpèdé

**Affiliations:** 1 Dermatology-Venereology, Faculty of Health, Cotonou, University of Abomey-Calavi, R. Benin; 2 Infectious Diseases, Faculty of Medicine of Parakou, University of Parakou, R. Benin; 3 Dermatology-Venereology Faculty of Medicine of Parakou, University of Parakou, R. Benin

**Keywords:** Giant condyloma, infant, electrocoagulation, Benin, West Africa

## Abstract

We report here a case of giant vulval condyloma in a two-year-old infant infected by her “baby sitter” without sexual abuse. Treated by surgical excision coupled with electrocoagulation, it was noted a rapid recurrence two weeks after treatment requiring a second electrocoagulation session. More than a year later, no lesion was noted, thus demonstrating therapeutic success. The unavailability of imiquimod in our context requires a systematic use of invasive treatment regardless of the age of the patient.

## Introduction

Adult’s anogenital condylomas are among the most frequent sexually transmitted diseases (STDs) [1]. These are benign lesions, associated in nearly 90% with human papillomavirus (HPV) genotypes 6 and 11 [2]. Studies have shown that oncogenic genotypes 16 and 18 may be associated with condylomatous lesions in nearly 12% of cases [3]. On the other hand, the sexual transmission of the ano-genital condylomata to children is much debated [4]. Clinically, it is not always easy to distinguish anogenital localization of warts from true condylomata acuminata (CA). Several studies have shown that sexual abuse is involved in 3 to 35% of cases of anogenital warts in children [5, 6]. The likelihood of sexual abuse increases with the age of the child. Thus, the positive predictive value of condylomas would be 36% between 4 and 8 years and 70% after 8 years [7]. The majority of anogenital condylomata in children would therefore be non-sexual transmission, whether by auto-inoculation, hetero-inoculation or through infected objects [5]. We report here a case of giant vulvar acuminate condyloma in a two-year infant noted without sexual abuse.

## Patient and observation

**Figure 1:**
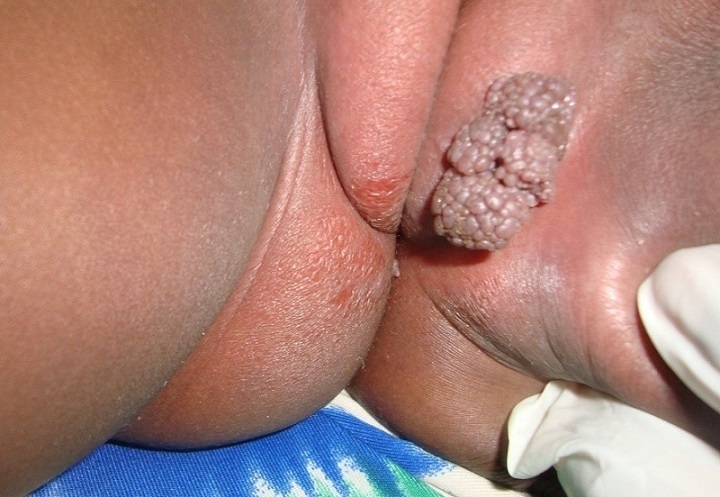
Giant vulvar condyloma

**Figure 2:**
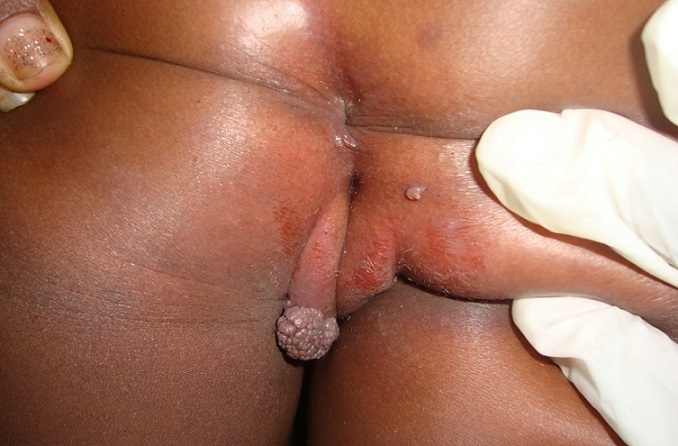
Condylomatous lesions of the anal margin associated with the giant condyloma

**Figure 3:**
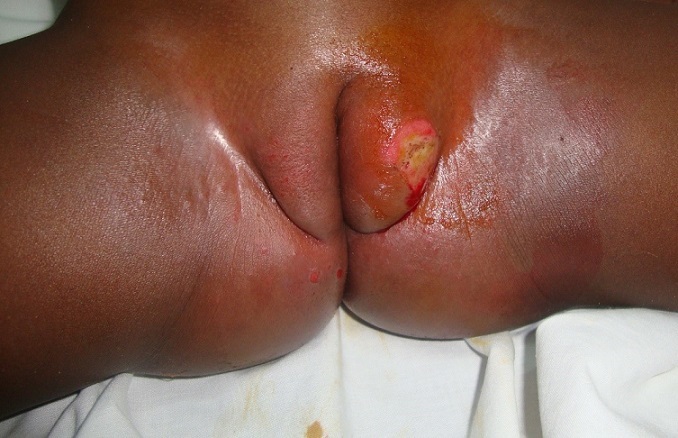
Immediate post-operative wound

**Figure 4:**
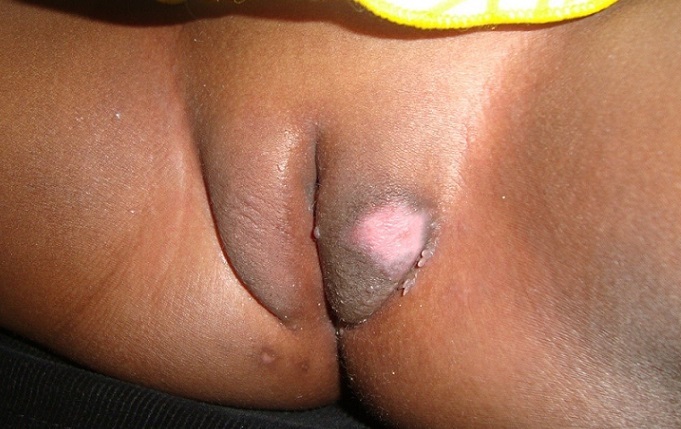
Two weeks after the first operation: recurrence

**Figure 5:**
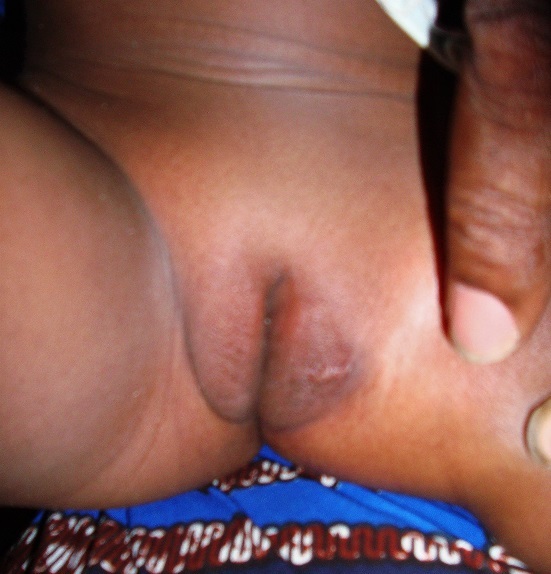
Six months postoperative: hypertrophic scar

**Figure 6:**
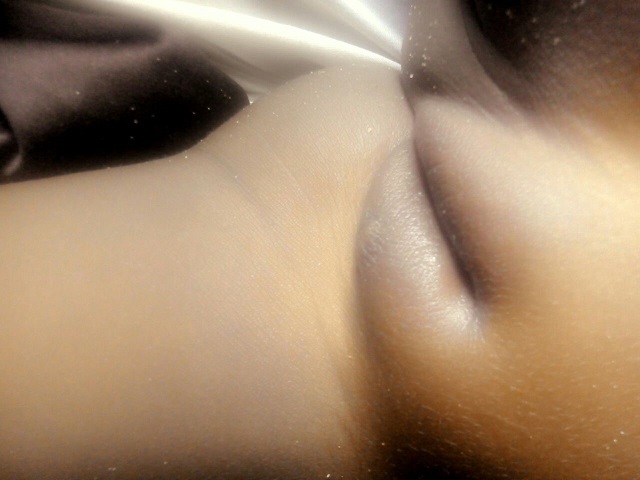
Eighteen months postoperative: good healing without recurrence

The case is about a female infant of two years brought into consultation in July 2015 by her mother because of a vulvar, asymptomatic lesions evolving for about 06 weeks, with a progressive and rapid increase in size. No prior treatment had been made before her admission. Clinical examination showed a vegetative tumor with a verrucous surface of 3 cm by 1.5 cm on the lower half of the cutaneous part of the large left lip (Figure 1). Examination of the anal margin found three tumor lesions of small size estimated in millimeter (about 1 to 3 mm), of a pinkish color, with a verrucous surface. They were the same lesions as that of the vulva but in miniature (Figure 2). Clinical examination of the rest of the tegument did not objectify any verrucous lesion; on the gynecological level, there was no sign of maltreatment (the hymen was intact, no obvious vulvar scar). In addition, the dermatological examination of her mother was showed no particular sign. An in-depth interrogation made it possible to identify the source person. It was the baby sitter in whom the clinical examination found multiple verrucous lesions of the hands and some condylomatous lesions of the pubis. Syphilitic and retroviral serology were requested in the infant and turned out to be negative. From a therapeutic point of view, despite the very young age of the patient and in the absence of alternative treatment, we carried out a surgical excision associated with electrocoagulation, under double local anesthesia (lidocaine cream and in subcutaneous injection) (Figure 3). The evolution was marked by a rapid recurrence in two weeks with the appearance of six small wart lesions located around the postoperative scar of the initial vulvar lesion (Figure 4). A second electrocoagulation proved mandatory. Six months later, the patient was reviewed and was observed to bear a vulvar hypertrophic scar without a condylomatous lesion (Figure 5). A local treatment with betamethasone ointment was established and nine months later, the hypertrophic scar disappeared (Figure 6). Thus, one and a half years after the second surgical operation, a complete cure was observed without any new recurrence.

## Discussion

Condylomas are subsequent to an infection of the keratinocytes by HPV, a DNA virus. In adults, transmission is essentially sexual. In children, three modes of transmission exist: perinatal (in utero and during delivery), horizontal (self- and hetero-inoculation known as “innocent”) and in sexual abuse [8]. Therefore, the discovery of condylomata acuminata (CA) in children requires a precise investigation in order to determine the mode of contamination and to rule out the hypothesis of sexual abuse. Children of all ages can develop CA, with a peak between zero and four years old. Girls are twice as often affected as boys [6]. Our observation well confirms this assertion. Giant condylomas in immunocompetent infants are rare. Their management is also problematic due to their young age and the therapeutic methods available, especially in Africa and most particularly in Benin where access to imiquimod and to liquid nitrogen is almost impossible. Skowron and al. [9] successfully treated a case of ano-genital CA in a 10-month-old boy with imiquimod in local daily application. Maha and al. [10] successfully treated a case of papular peri-anal condylomas of the infant with 5% salicylated vaseline. The rapid recurrence observed in our patient was probably related to subclinical lesions prior to the first surgical procedure. The literature confirms this hypothesis because it has been shown that most therapeutic methods used in adults (cryotherapy, laser vaporization, electrodestruction, salicylic acid, surgical excision, podophyllotoxin) are associated with high rates of recurrence through persistence of infected cells with HPV around visible lesions [11]. With our patient, the contamination was by hetero-inoculation (by her baby sitter). It was certainly a transmission through her hand during the child´s toilet or through various objects. This poses a public health problem because most modern households have female servants to care for children. In view of this mode of contamination of HPV, it is necessary to make our populations aware of the danger or risk involved if we are not in the same time concerned about the health of our servants. Adégbidi and al. [12] in Benin published a first case of condylomata acuminata in a 16-month-old boy in which a sexual abuse by the domestic was strongly suspected without any formal evidence. Dahmani and al. [13] published in Algeria, recently in 2016, a case of ano-genital warts in an 11-year-old girl following sexual abuse.

## Conclusion

The interest of our observation lies in the rarity of giant condylomas in infants, the therapeutic method used successfully, the absence of recurrence after more than one year, the non-sexual contamination of these condylomas in an immunocompetent patient. Moreover, this study shows the necessity to make available the therapeutic means less or not invasive for the management of these cases. It is also important to ensure the medical supervision of house workers because the health of the whole family depends on it.

## Competing interests

The authors declare no competing interests.
